# Navigating complexity of child abuse through intuition and evidence-based guidelines: a mix-methods study among child and youth healthcare practitioners

**DOI:** 10.1186/s12875-020-01226-6

**Published:** 2020-08-01

**Authors:** Jetske C. Erisman, Kevin de Sabbata, Teun Zuiderent-Jerak, Elena V. Syurina

**Affiliations:** Athena Institute, Faculty of Science, Vrije Universiteit Amsterdam, De Boelelaan, 1085 1081 Amsterdam, HV The Netherlands

**Keywords:** Intuition, Gut feeling, Child and youth health care, Child protection, Social work practice, Child abuse, Guidelines, Evidence-based medicine, Decision-making

## Abstract

**Background:**

Dutch child and youth health care (CYHC) practitioners monitor and assess the well-being of children. One of their main concerns is identifying cases of child abuse, which is an arduous and sensitive task. In these contexts, CYHC-practitioners use both evidence-based guidelines aimed at increasing the quality of care through rationalised decision-making, and intuition. These two practices are seen as being at odds with each other, yet empirical research has shown that both are necessary in healthcare. This study aims to unravel how intuition is perceived and used by Dutch CYHC-practitioners when identifying and working with cases of child abuse, and how this relates to their evidence-based guidelines.

**Methods:**

A sequential exploratory mixed-methods design: in-depth semi-structured interviews with CYHC-physicians focused on perceptions on intuition, which were followed by a survey amongst CYHC-practitioners on the recognition and use of the concept.

**Results:**

The majority of CYHC-practitioners recognise and use intuition in their daily work, stating that it is necessary in their profession. CYHC-practitioners use intuition to 1) sense that something is ‘off’, 2) differentiate between ‘normal’ and ‘abnormal’, 3) assess risks, 4) weigh secondary information and 5) communicate with parents. At the same time, they warn of its dangers, as it may lead to ‘tunnel vision’ and false accusations.

**Conclusion:**

Intuition is experienced as an integral part of the work of CYHC-practitioners. It is understood as particularly useful in cases of child abuse, which are inherently complex, as signs and evidence of abuse are often hidden, subtle and unique in each case. CYHC-practitioners use intuition to manage and navigate this complexity. There is an opportunity for guidelines to support reflection and intuition as a ‘good care’ practice.

## Background Intuition or evidence in child and youth health care?

The World Health Organisation reports that, worldwide, one in four adults were physically abused as children [1]. This widespread, complex, and sensitive issue requires careful responses from child welfare organisations. Signs of abuse can be difficult to detect, hidden, and multi-interpretable, and identification processes are reliant on the personal judgement of practitioners [2–5]. The definitions of ‘abuse’ or ‘neglect’ are themselves contested, as some aspects have blurred boundaries in respect to moral, cultural and contextual interpretations of ‘healthy families’ [6–8]. Owing in part to these inherent complexities, many cases of child abuse are suspected to be missed every year [6, 7, 9].

Child abuse can be dealt with by a range of different professional infrastructures. In the Netherlands, it is largely managed by the child and youth health care (CYHC) system.^1^ CYHC-practitioners are tasked with monitoring and assessing the mental, social and physical well-being of all children from birth to age 19, through regular, voluntary visits to CYHC-centres, schools and homes. When CYHC-practitioners encounter signs and evidence of abuse, they can suggest support services for the family or, in severe cases, refer the family to other child protection institutions for investigation into further support or (legal) intervention [10].

In identifying child abuse, CYHC-practitioners face challenges, such as high workloads, time pressure and having to navigate the different perspectives of multiple actors [8, 11, 12]. Their work is further challenged by a lack of observable evidence, while the amount of information their decisions must take into account is vast [11]. Fuelled by public inquiries and attempts to modernise the field - comparable to the upsurge of evidence-based medicine in other care domains - recent movements have focused on standardising practices through evidence-based guidelines and instruments [11, 13]. CYHC-practitioners use informal skills or knowledge practices alongside guidelines and instruments to make sense of their cases [11, 13, 14]. One of the most prevalent, yet ambiguous, mechanisms of informal knowledge practices is *intuition* [4, 8, 15, 16], which is commonly contrasted with attempts to make practice more evidence-based [17, 18].

This study aims to unravel how intuition is perceived and used by Dutch CYHC-practitioners when identifying and working with cases of child abuse, and how this relates to their guidelines. The study is guided by the research question: *How do CYHC-practitioners understand the role of intuition and its relation to evidence-based medicine in their work related to cases of child abuse?* The conducted research consists of in-depth, explorative and semi-structured interviews with Dutch CYHC-physicians on their perspective on the use of intuition in the case of child abuse, and a survey on the recognition and use of intuition, which was spread amongst CYHC-practitioners (physicians, assistants and nurses). This article argues that intuition is a *practice* which allows CYHC-practitioners to manage and navigate the complexity involved in their cases, evidence and communications.

### Child abuse guidelines and informal knowledge practices

When CYHC-practitioners investigate suspected cases of child abuse, multiple complexities converge [19, 20]. On the one hand, there are different notions and definitions of ‘abuse’, ‘safety’ and ‘healthy’, but also of what a ‘family’ is and should look like [8]. On the other hand, CYHC-practitioners must deal with additional complicating factors: shortage and large turnover of staff, a heavy workload, time-pressure and needing to work together with many different organisations and actors [11, 13, 20]. In grasping the situation and identifying whether children are at risk, CYHC-practitioners need to make sense of all these different realities and engage in *coordination work* to navigate the complex situation and make decisions on what a family needs [21]*.*

Acknowledgement of complexities in identifying child abuse has led to attempts at modernising the field and reducing uncertainties of practice through the provision of standardised assessments, guidelines and instruments [13, 16], a trend which can also be seen in the Netherlands. In the investigation of suspected cases of child abuse, CYHC-practitioners are supported by evidence-based guidelines, which aim to improve the quality of care and preventative practices [22, 23]. These guidelines emphasise the importance of collecting factual evidence, on which CYHC-practitioners ought to base their decisions [2, 16]. Broadhurst et al. (2010) state that this trend towards standardised guidelines leaves less room for soft or informal skills, which are considered as highly prevalent and useful in practitioners’ handling of complexity and uncertainty [13, 18, 24–26]. As the importance of these skills is acknowledged, Dutch clinical guidelines have begun to include informal knowledge practices, mainly being the use of intuition [27–29].

According to the Dutch guidelines for child abuse, CYHC-practitioners should “develop their intuition well” (p.84), but “avoid basing decisions on it” (p.71), and “check their intuition with factual evidence” (p.81) [2]. The guidelines thus appear to transmit a rather ambiguous message, with explanations of *what* intuition is, *how* to develop it and *how* to use it in practice lacking. The advice on intuition in the Dutch guideline is based on an article by Munro (1999), who describes intuition as fast decision-making that is on a spectrum with rationality. She warns of unguided intuitive decision-making in child care, which is prone to error and bias, as CYHC-practitioners often encounter unreliable evidence [30]. In order to support CYHC-practitioners in the detection of child abuse and their decision-making, there is a need for research on the use of intuition in practice and how this can be supported by guidelines.

### Intuition

While there is consensus on the existence and usefulness of intuition in many fields, this is not the case for its definition [17]. Brenner and Tanner [31] were among the most prominent authors to state the importance of intuition in nursing. They stated that intuition was considered inferior to statistical, technological and numerical data, and deemed too feminine, mysterious and subjective to be included in formal logic or rational calculation. They aimed to demystify intuition and argued that it is “understanding without rationale” (p.23), formed by expertise and relying on quick and unconscious clinical observation: a form of expert knowledge [31]. This was critiqued in light of the authors’ apparent unwillingness to dissect the concept of intuition, which Pellegrino [32] argued is a ‘moral and intellectual responsibility’ [32] (p.187). Lamond and Thompson [33] agree, stating that patients have the right to know how their diagnosis is made and that healthcare practitioners should be able to legitimize their decisions through greater transparency. At the same time, they acknowledge the difficulty of the complex and “messy” problems that practitioners encounter, and the use of intuition in such cases.

The abovementioned calls for transparency have led to attempts at disentangling intuition. Welsh and Lyons [34] state that intuition has a basis in both tacit and formal knowledge. Tacit knowledge is seen as implicit knowledge that is obtained by experience in the field, while formal knowledge is the knowledge gained by training and education. They aim to demystify the concept by arguing that experienced healthcare practitioners gain a quick and holistic overview of a patient’s or client’s situation and its potential red flags. In line with this definition, Dutch general practitioner and researcher Stolper [35, 36] has ascribed the mystical element of intuition to the bodily sensation of ‘gut feeling’. It is one’s gut giving a warning or judgement of a patient’s situation, in contexts where evidence lacks and forming a rational conclusion is not possible. Studying gut feeling in general practitioners, Stopler found it to derive from both a doctor’s contextual knowledge of the patient and their medical knowledge, linking gut feeling to expertise [35, 37].

Sadler-Smith [15] argues that intuition as expertise does not cover the concept and the friction it brings in practice, suggesting a definition of intuition as: “An involuntary, difficult-to-articulate, affect-laden recognition or judgement, based upon prior learning and experiences, which is arrived at rapidly, through holistic associations and without deliberative or conscious rational thought” (p.31), a definition more closely linked to intuition as a mode of thought [15].

Recent studies have started to disentangle intuition further. Instead of broadly defining the concept, these orient more on the different elements of intuition and the characteristics of its users. Ruzsa, Szeverenyi and Varga [25] studied healthcare practitioners in different medical specialities and conducted surveys to see whether person- or job-specific factors contributed to their use of intuition. They concluded that high emergency, complexity and responsibility led to increased use of intuition. In addition to the raised utilisation of intuition in response to high levels of emergency and complexity, the study found that experienced practitioners used intuition more than their novice colleagues [25], while other studies found no link between experience and use of intuition [24, 38]. All studies describe the need for a better understanding of intuition and factors or characteristics that may influence it.

In short, the debate on intuition has transitioned through several phases: first, there was a need for acknowledgement of the concept, followed by a focus its disentanglement and comparison to other ways of thinking. Next, the discussion turned to developing a better understanding of what intuition is influenced by and who uses it. Most recently, there has been a turn towards understanding *how* intuition is used. In this article, we focus on the latter and follow the line of Sadler-Smith [18] in trying to understand intuition as a practice. We argue that intuition can be seen as a situated ‘knowledge practice’ in which knowledge is created, aligned and weighted [15, 21]. By shifting the focus to practices, we can begin to explore how intuition is used in complex situations.

## Methods

As previous studies on the topic are limited, an exploratory sequential mixed-methods design was deemed appropriate [39]. First, qualitative in-depth material was gathered through interviews with CYHC-physicians. The interviews were coded through an iterative open coding process, from which three main themes emerged. These themes were used to create an exploratory survey, which was distributed to collect supportive quantitative data amongst other CYHC-practitioners (nurses, assistants and other CYHC-physicians). This allowed insights into the recognition and use of intuition amongst other CYHC-practitioners.

### Qualitative data

The qualitative material consisted of 14 semi-structured interviews with CYHC-physicians in the Netherlands, conducted between May and July 2017.

#### Participant recruitment and data collection tool

Interviewees were recruited through emails sent to different CYHC-practices in the Netherlands. After the initial email contact, interviews were arranged by telephone. The interview guide was tested in four pilot interviews with CYHC-professionals, after which changes were made in terminology used and in the order of questions. The interview guide consisted of three themes: after an introduction, participants were asked to express their associations with ‘intuition’ in relation to their work,^2^ followed by questions on their definitions and use of it and whether they could recall a case in which they used it. Then, interviewees were asked about their experience with cases of (suspected) child abuse and the guidelines and instruments available to support them. Lastly, they were asked about the use of intuition in relation to these guidelines and instruments, as well as in their decision-making processes. An iterative approach to interviewing was used, which allowed new themes to arise and enabled member-checking. To increase the amount of available data and to further strengthen the methodological rigour, interviews were collected by two interviewers. Qualitative data collection ended after data saturation was reached.

On average, interviews lasted 1.5 h. Most interviews took place in offices, meeting rooms or consultation rooms in CYHC-practices, with two exceptions: one interview took place in the home of an interviewee and the other in a cafe. The interviewees worked at CYHC-practices throughout the Netherlands, and their work experience within the CYHC-system ranged between 6 and 37 years. Twelve out of 14 interviewees were working as CYHC-physicians, one as a nurse and policy advisor for child abuse issues, and one as an assistant of a CYHC-physician. Four interviewees were male and the remaining 10 were female. All interviews were conducted in Dutch.

#### Data analysis

The recordings of the interviews were transcribed *verbatim*. To ensure member-checking and improve rigour, all transcriptions were summarized, and both the full verbatim transcription and the summary were sent to the informants for comments and feedback. None of the informants asked to make changes. When the project ended, all participants received a summary of the findings, which allowed for another round of member-checking. Some participants asked questions based on the summary, which were used to clarify the results section.

The transcriptions and field notes of the interviews were analysed through Braun and Clarke’s thematic analysis (2006), using ATLAS.ti 7.5.18. First, all material was read through to gain an overview of the material, then, each transcript was coded through open coding by JE. All codes were checked for overlap and nuances, after which they were grouped into themes. This was an iterative process led by JE and discussed and cross-validated within the research team until consensus was reached [40]. The major themes in both interviews and the survey are used to present the data in the results section, namely: 1) possible definitions of intuition in decision-making in the CYHC-system, 2) attitudes of CYHC-practitioners towards it, and 3) the way in which CYHC-practitioners use intuition in cases of child abuse. These themes formed the basis of the survey. An overview of our codes can be found in: Additional file 1 - Overview of codes.

### Quantitative data

The quantitative data was collected through a web-based exploratory survey using Qualtrics XM, focusing on the recognition, use of and attitudes towards intuition of Dutch CYHC-practitioners.

#### Data collection tool

After deliberation with interviewees and the research team and based on the validated translations of existing surveys on the topic in Dutch and English [37, 41, 42], it was decided that ‘gut feeling’ would be used in the survey, but that its definition would include elements of other terms and would mention intuition: *‘Gut feeling is the intuitive feeling that something is right or wrong, without any obvious reasons for it being found (directly)’*.

The self-administered survey consisted of several elements. First, an introduction into the study and the topic was given, followed by general demographic information. Next, each respondent was asked whether they recognise gut feeling in their job as a CYHC-professional. If the respondent stated that they did not recognise it, they were directed to seven Likert-scale questions focused on the recognition of gut feeling. If the respondent did recognise it, their set of 11 questions focused on the use of attitudes towards gut feeling, which asked about trusting in their gut feeling, the subjectivity of gut feeling, and whether they trusted colleagues who use it. The survey questions can be found in Additional file 2: Survey questions.

The survey was tested in three rounds. First on masters students at the VU University Amsterdam, then on members of the research team, and lastly, on 10 CYHC-physicians. The survey was revised and adapted after each testing round. Adaptations were made on the wording used and definitions given, which ensured clarity and correctness of the questions asked.

#### Sampling and data analysis

Included in the survey were registered CYHC-practitioners who were fluent in Dutch and who had experience with (suspected) cases of child abuse and neglect. A link to the survey and an elaborate information letter were sent to 25 Dutch municipal healthcare practices (*Gemeentelijke Gezondheidsdienst*) via email. Data was collected between June and July 2017.

In total, 339 Dutch CYHC-practitioners accessed the survey. Ten respondents did not fill in any questions and were not included in the final dataset, which consisted of 329 responses. The percentage of missing data in the total dataset was 2.09%. It was under 5% for each variable and found to be random through Little’s MCAR test. Most of the respondents were female (93%) and worked as nurses (59.6%) (*n* = 329). Their years of experience ranged from 0 to 40 years (*n* = 303, mean = 14.96, standard deviation = 10.2), of whom 78.9% had over 5 years of experience. An overview of the characteristics of the survey participants can be found in Table 1. The data was analysed by conducting descriptive statistics using IBM SPSS Statistics for Windows version 26.0.0.0. The analysis was conducted by JE and ES, in consultation of a statistician.

**Table 1:** Sample characteristics survey ‘Decision-making process child and youth health care system’

**Gender (*n*** **= 329)**	**%**	**N**
Female	93	306
Male	6.1	20
Other	0.3	1
No answer	0.6	2
**Profession (*n*** **= 329)**	**%**	**N**
CYHC-physician	30.4	100
Nurse	59.6	196
Assistant	6.7	22
Assistant specialised in infants	2.7	9
**Years of experience (*n*** **= 303)**	**Number of years**	
Minimum	0	
Maximum	40	
Mean	14.96	
Standard deviation	10.20	

#### Reliability and validity

To explore the factorial structure of intuition/gut feeling amongst CYHC-practitioners, nine items were subjected to an exploratory factor analysis with orthogonal varimax rotation. These nine items form the Likert scale questions for respondents who recognise intuition/gut feeling, and the set of questions for participants who did not recognise intuition/gut feeling did not generate enough responses to be tested (*n* = 5). The Kaiser-Meyer-Olkin measure verified the sampling adequacy for analysis (KMO = .748). The Bartlett’s Test of Sphericity was tested resulting in Chi-Square value 828,359, *p* < 0.001 and was therefore deemed acceptable for factor analysis. This resulted in two factors accounting for 56.7% of variance: ‘Attitudes’ and ‘Use’. The factor *Attitudes* was comprised of four items reported on a five-point Likert scale that explained 33.3% of the variance, with factor loadings from .746 to .815, using a cut-off point of .40 and Eigenvalues over 1. Internal consistency was tested with Cronbach’s Alpha, resulting in α = .772, reflecting good reliability. The factor *Use* consisted of five items reported on a five-point Likert scale, which explained 24.3% of the variance. Factor loadings ranged from .603 to .766. Cronbach’s Alpha was α = .765, again reflecting good reliability.

## Results

Three main themes emerged from qualitative data analysis, which formed the basis of quantitative data collection and the order in which the results will be presented: 1) the recognition and possible definitions of intuition in decision-making in the CYHC-system, 2) attitudes of CYHC-practitioners towards intuition and 3) different ways in which CYHC-practitioners use intuition in cases of child abuse. The outcomes of both data collection processes will be presented in an integrated manner.

### Recognition and definitions of intuition

The results of both types of data collection suggest a high recognition rate of intuitive feelings by CYHC-practitioners: all interviewees and 96.7% (*n* = 329) of survey participants stated that they recognise and experience this intuition or gut feeling in their daily work, of whom 48% stated that they experience it ‘sometimes’ and 30.1% experiences it ‘often’ (*n* = 326). All interviewees recognised and experienced intuitive feelings:

*Yes, [I recognise intuition] in the sense that you try to make an assessment of a situation. And because of the questions you ask and the answers you get, you naturally get a bit of an idea on whether what you're told is correct, or not. And you can’t always put your finger on it, so you call it intuition (CYHC-physician, 17 years of experience*)*.*

Even though they recognised intuition, the majority of interviewees struggled to define these feelings, suggesting that it is personal and influenced by experience:

I would describe intuition as a feeling with which you make decisions that are based on experience. So, it may be something unconscious, but it is secretly something conscious and based on previous experiences (CYHC-physician, 35 years of experience)*.*

The abovementioned quote links intuition to experience. Interviewees also linked intuition to implicit knowing, assessment of situations and decision-making processes:

Intuition sounds as if you are guessing or something, while I think that intuition plays an important role. I would prefer to describe it as a sort of sensitivity, rather than intuition. You pick up a lot of signs that give you a certain feeling (CYHC-physician, 15 years of experience)*.*

Definitions and opinions on intuition differed, but most interviewees experience it as an uncontrollable feeling or sensation that occurs regularly and originates from the senses: “*I think that your intuition is always on, even when you don’t want it to be. You see, hear and smell things and you form an image right away” (CYHC-physician, 32 years of experience*)*.* All interviewees agreed that intuition stems from signs that they picked up, for example smells, verbal and non-verbal communication; or from stories they have been told by others in the network of a family.

### Attitudes towards the use of intuition

**Fig. 1:**
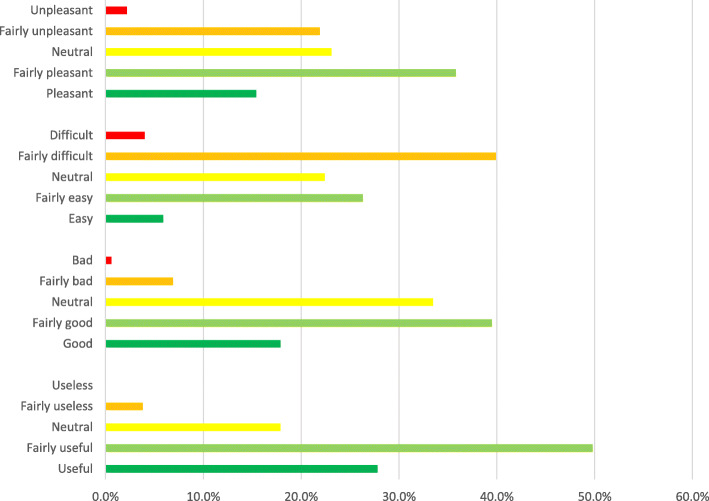
Survey responses regarding the use of gut feeling. *n* = 319

As participants generally acknowledged the concept of intuition, the next step was to look at the attitudes towards intuition and intuitive decision-making. The survey measured these attitudes using Likert scales, of which the responses are summarized in Fig. 1.

Respondents mainly considered it to be ‘fairly useful (*nuttig*)’ (49.8%, *n* = 319), ‘fairly difficult (*moeilijk*)’ (39.9%, *n* = 323, ‘fairly good (*goed*)’ (39.5%, *n* = 319) and ‘fairly pleasant (*prettig*)’ (35, 8%, *n* = 324). These responses show ambivalences towards intuition: it is experienced as useful and good, yet it is difficult and not everyone enjoys using it. This also became apparent in the interviews. Often, a question of intuition prompted an initial positive response, after which CYHC-practitioners expressed their concerns. All participants affirmed that steps need to be taken when intuition is sensed: *“Certainly, you are obliged to do something with [intuition], otherwise you are negligent. [ …*] *To me, that is the most important thing. Otherwise you ignore your duty of care. After all, I’m a doctor for a reason” (CYHC-physician, 25 years of experience*)*.* When asked what action needs to be taken, they responded that they look for facts, as mentioned in the guidelines:

What you try to do is to get rid of that gut feeling as quickly as possible and replace that intuition with facts. Because my feeling says it's not quite right, but which questions should I ask to check that? [ … ] Is my intuition incorrect? So [intuition] is a bit like your compass in the conversation (CYHC-physician, 17 years of experience)*.*

CYHC-physicians thus aim to ‘get rid’ of intuition by checking it with facts and state that ignoring intuition would be negligent. When asked about these ‘facts’ and what they consist of, CYHC-physicians mentioned that they are difficult to determine and to define. They stated that facts could be tangible elements such as a *“black eye or clothes that are too small” (CYHC-physician, 14 years of experience)*, but that ‘facts’ are often constructed when comparing narratives of the people involved in the (suspected) case, such as the family themselves, the children’s teachers, trainers, GPs or other healthcare practitioners who interact with the family on a regular basis. These people form the *“eyes and ears of CYHC-physicians” (CYHC-physician, 14 years of experience)* and can deliver input to test the factual soundness of intuition.

In working with intuition, *ignoring* it is thus seen as dangerous, but interviewees also stated that it is unsafe to blindly *trust* intuition, as it may lead to narrow-mindedness and missing signs. Survey respondents who did recognise gut feeling (*n* = 318) were asked whether they felt that they can trust their gut feeling when making decisions, in which ‘decisions’ were defined as: *“the decision to plan a follow-up appointment, to wait or to discuss the case with a colleague”.* Most respondents answered that they could trust it (41.7%, *n* = 314). One interviewee explained that blindly trusting intuition can lead to ‘tunnel vision’: focussing on one explanation of a situation, without taking other possibilities into account. According to the interviewee, this is dangerous as it may damage the relationship with parents, which could lead to misinterpretations and false accusations of child abuse, having major implications for both the families and practitioners involved.

That’s what makes it [intuition] dangerous, if you are convinced it is neglect or abuse, well, see what happens to your body language: you’ll only focus on proving that these parents are no good. For example, because of your approach, the parent will start stumbling, they will lean backwards and that only confirms your thoughts: something is wrong here. Once this happens, you’ll never be able to have a good relationship with this parent anymore. (CYHC-physician, 32 years of experience)*.*

CYHC-physicians aim to avoid tunnel vision by discussing all their cases with peers, nurses and their assistants. To get a sense of the general perception on intuition in CYHC-practices, interviewees were asked whether they thought their colleagues use intuition, to which one replied that they *“hope they do: otherwise you won’t be able to function” (CYHC-physician, 9 years of experience*)*.* They explained that the focus on communication and ‘social medicine’ in the CYHC-system made the use of intuition vital:

I do think that CYHC-physicians are more sensitive and more social than the average doctor or surgeon. It might be due to how your education raises you and what is allowed in your profession. We talk a lot about feelings and the personal lives of people, so it has a place in our profession. [ … ] So generally speaking, we are more sensitive people (CYHC-physician, 15 years of experience)*.*

Intuition is experienced as an integral element of the daily work of CYHC-practitioners, and not as a cause of friction with colleagues or guidelines. Despite this, Dutch guidelines for CYHC-practitioners on child abuse discourage decisions based on intuition. When interviewees were asked whether they knew what the guidelines said about intuition, the majority stated that it was not mentioned, but they also stated that they did not regard the guidelines as a useful tool as it was seen as too lengthy and generalised for their specific cases. They explained that child abuse is complex and personal, which requires a subjective approach that they felt was not captured in the current national guidelines.

### Using intuition

In order to explore the use of intuition, survey respondents who recognised intuition or gut feeling (*n* = 318) were asked whether they are allowed and enabled to use intuition, to which 48.1% (*n* = 314) responded affirmative. Knowing that feelings of intuition or gut feeling are experienced and used by CYHC-practitioners in their decision-making process in the case of (suspected) child abuse, the interviewees were asked how they use these feelings. It was found that intuition can arise in different stages of the decision-making process and interviewees mentioned five distinct levels of working with intuition in their daily work: 1) to sense that something is ‘off’, 2) to normalise deviant or uncommon behaviour, 3) to assess risk, 4) to weigh secondary information and 5) to communicate with parents or caretakers.

The first level of the use of intuition is to sense that ‘something is off’. As one of the interviewees stated: *“If you are doing an examination, or look into their [the child’s] development, even if they meet the criteria, there is something that makes me think: something is off. Even though they do just as well as the children who come before them and after them, still there is something that worries you. That’s intuition” (CYHC-physician, 35 years of experience).* As mentioned by this informant, the source of concern can be unclear, but the sense of ‘something being off’ was often linked to intuition by informants.

The second level is that intuition is used to differentiate between ‘normal’ and ‘abnormal’, in which interviewees subdivided abnormality in ‘abnormal cases that can be normalised’ and ‘dangerously abnormal cases’. Normalities and abnormalities become more challenging when CYHC-practitioners work with people with different backgrounds. When actors external to the family circle, such as school teachers, share their concerns about the well-being of a child, they communicate using their own norms and values, which can differ from those of the family. CYHC-practitioners juggle their own norms and values, those of society and the opinion that they are expected to have as a CYHC-practitioner:

Dealing with different cultures makes you act differently, whether they are refugees or not, or just people with different ways of behaving. I think norms and values are very important in our profession and sometimes you have to set them aside and not judge people. Communicating with refugees is more difficult, so then you’ll have to trust your intuition even more (CYHC-physician, 9 years of experience)*.*

When CYHC-practitioners deal with families with different ideas of normality, regardless of background, they have to make a decision on whether a situation is to be considered dangerous or risky for a child, or not. When there are risk factors or dangerous elements in a family situation, CYHC-practitioners need to make decisions on next steps that need to be taken. This leads to the third use of intuition: the assessing of risks and the ability of the family to cope or solve problems. *“The role of intuition is: the moment I see a mother with a baby who cries a lot, I have to assess whether the mother is able to cope with this or not” (CYHC-physician, 37 years of experience).* CYHC-practitioners assess the urgency of a case, in order to decide what kind of care they have to arrange for a family. Interviewees stated that intuition is fast and useful in this process.

The fourth level is that secondary information is weighed with the use of intuition. CYHC-practitioners do not only rely on information and signals provided by the child and their families, but also on information and narratives coming from others in the environment of the child, such as teachers, sport coaches or general physicians. Based on their opinions and stories, CYHC-practitioners aim to construct a truthful image of the child’s situation and decide whether it is considered to be harmful or not.

*To solely make objective observations is very difficult, because we always interpret, we aren’t objective. And if so, then you’ll get some facts, some signs, and how will you measure those? I don’t think you could do that without intuition. Or when you have to decide whether people tell the truth or not: if you have to judge someone on their words, you won’t be able to do that without intuition (CYHC-physician, 32 years of experience*)*.*

CYHC-practitioners use intuition to sense which statements are truthful and to reconstruct a complete picture of the situation. As child abuse is often hidden and occurs behind closed doors, CYHC- practitioners rely on the information of others and consensus amongst those others as to what is occurring in the family. They gather information from the family members themselves, their files and the stories of people who are working closely with the subjects. As one of the interviewees described, after sensing that there might be something wrong or that she feels like there is a risky situation, she will start gathering additional information, mainly by asking more questions to everyone involved. She wants to ensure that there are no gaps in the information or friction between different sides of the story, as they can be clues to something being wrong (CYHC-physician, 25 years of experience). When CYHC- practitioners realise that someone is not telling the (full) truth, or they find friction or different interpretations in the narratives of the people involved, it is seen as a sign to raise alarm. Within evidence-based medicine, is it often assumed that uncertainty can be eliminated by gathering more information [27, 43, 44]. However, interviewees state that while gathering more information is their first response to uncertainty as well, the irregularities or uncertainties they find when triangulating narratives are embraced as evidence as well. CYHC-practitioners use their intuition to weigh the incoming information and to eliminate or embrace the uncertainty that follows.

Finally, intuition is used to communicate with parents and to negotiate their ideas or solutions. Each family and each case of suspected child abuse is unique and CYHC- practitioners need to sense how they can work with the different actors involved. As communication is: *“the only tool CYHC-physicians have” (CYHC-physician, 32 years of experience)*, they need to know how to use it. Trainings are focused on conversational techniques, but interviewees said that it is not only techniques, but that it is also necessary to ‘feel’ the situation in order to know what to do. Interviewees mentioned intuition as a mean to sense this: *“That’s also intuition, that you hear something that makes you think: wait, stop, I have to ask about that” (CYHC-physician, 35 years of experience).*

The interviews show that intuition plays an influential role within decision-making in cases of (suspected) child abuse. CYHC-practitioners use intuition to assess and judge a situation and to communicate their opinion with colleagues and with the family. Intuition becomes a practice, rather than a feeling, through which CYHC-practitioners can find issues and discuss them with parents or carers of children. It helps them to identify signs of abuse and to assess and communicate this, allowing practitioners to act upon signs earlier and discuss it appropriately with parents/carers and others involved. This is important, as early detection and discussion of problems saves both parents and children from harm or severe consequences.

## Discussion

The results of this study show both the difficulty of articulating and utilising an abstract concept like intuition, as well as an uniform agreement about its high potential for decision-making in the practice of child and youth health care. Whilst quantitative data shows that most CYHC-practitioners do recognise and use intuition, the qualitative data articulates *how* intuition is conceptualised, understood and enacted amongst other knowledge practices.

Our findings reflect the results of similar studies and articles on informal knowledge practices in CYHC-systems or social work [11, 13, 25], such as Ruzsa, Szeverenyi and Varga [25] who showed that in situations with high levels of complexity, uncertainty or emergency, healthcare practitioners are more likely to use intuition. Our participants explained that they encounter complexity and uncertainty in all stages: the topic is sensitive; they lack sufficient time to thoroughly ‘get to know’ a family and are forced to make moral decisions. According to Broadhurst et al. [13] and Saltiel [11], this explains why informal knowledge practices are inherent to social work, which is similar to the CYHC-system. Even though the guidelines ask for facts and evidence, CYHC-practitioners mainly work with narratives of the people involved. Within these conversations, they look for friction between the recollections or gaps in their knowledge of the family. Narratives, friction and gaps are then used as facts to support their case, which also shows that the ‘evidence’ and ‘facts’ used in the decision-making process are highly complex. CYHC-practitioners manage this multi-levelled complexity with intuition, allowing them to take fast, yet well-informed decisions [8, 45].

While our study suggests that there is a positive stance towards intuition, it also displayed the multiple aspects of intuitive feelings, which provides an insight in its complexity: participants recognise it and find it useful to a certain degree, but they also experience it as a difficult feeling to deal with. Interviewees noted the difficulties of using intuition and warned of its danger when not investigated or handled reflexively, which is a concern shared by Benner and Tanner [31]. Interviewees and survey responses showed that intuition is, paradoxically, seen as integral to carrying out duties legitimately but also risking the legitimacy of the practitioners’ actions when intuition is trusted ‘blindly’.

The ambiguous stance towards intuition and call for reflexivity indicates that it can be a helpful practice in decision-making, but also highlights the need for a situated understanding of intuition and guidance on how to use it as ‘good care’ [46]. Notions of ‘good care’ are starting to change and consequently there is a need to connect evidence-based approaches to reflexive practices that help find fitting solutions for individual problems [29, 44]. Within this new school of thought on good care, uncertainty is seen as inherent to clinical problems and encounters. An example on how this can be done can be found in an ethnographic study on integrating the process of diagnostic work in a guideline for problem behaviour in elderly care [44], in which the researchers shifted the focus of the guideline towards asking questions and enabling conversation and consensus amongst the different healthcare practitioners involved in the care of a person. The guideline included a nine-question list that forced practitioners to reflect, discuss and think outside the box. Their response to this new type of guideline was good, as it assisted them in improving care for their patients. A similar style guideline could help CYHC-practitioners in avoiding tunnel vision when using intuition and guide them further in conversation and triangulation. When intuition is supported as a situated practice in the CYHC-system and practitioners are taught how they can use it, it could contribute to earlier detection and prevention of child abuse cases.

### Limitations and further research

The main limitation of this study lies in the survey. The wide variety of answers in some questions and the difficulty of creating questions that ‘measure’ gut feeling illustrates the complexity of the concept. The quantitative survey was not designed to have a total score that would measure the overall concept of ‘gut feeling’, which was confirmed by several tests, including correlations and multiple regression analysis with insignificant results. Knowing these limitations, only descriptive statistics were used. In further research, we suggest the validation of a survey directed to practitioners in the CYHC-field or social work, which can serve to attain a deeper understanding of elements that could influence the use of intuition or gut feeling, such as someone’s position in medical hierarchy or the backgrounds of practitioners.

Another complicating factor in this study was terminology. Participants had different personal definitions of both intuition and gut feeling, as well as other related concepts. To gain an understanding of how practitioners perceive intuition, the interviews were focused on unpacking this by returning to the meaning and use of it several times. Moreover, all interviewees received a summary and a verbatim transcript of their interview and were given the opportunity to reflect upon this or make changes if they deemed necessary. Throughout the study, it became apparent that connotations and past definitions play a major role in the individual ideas of intuition, but also in policy-making and structural levels. It would be of great use to conduct a linguistic study into the changing perceptions on intuition.

## Conclusion

This study aimed to understand how child and youth healthcare-practitioners perceive the role of intuition in their work and in relation to evidence-based medicine, in the case of child abuse. The findings show intuition is widely recognized and used amongst practitioners in the field and that CYHC-practitioners view intuition as a practice that is inherently part of their role, in which formal knowledge, experience and personality are combined. Intuition is stated to be particularly useful in the highly complex area of child abuse, where signs and evidence are subtle and unique in each case. CYHC-practitioners use intuition on five distinct levels through which they navigate the complexities of suspected child abuse: to sense that something is ‘off’, to differentiate between ‘normal’ and ‘abnormal’, to assess risks, to weigh secondary information and to communicate with families. This shows that intuition is not opposite to nor on a spectrum with evidence-based medicine, but that practitioners practice both in order to provide and improve care for their clients. For guidelines, this means that they ought to not only aim to include different types of knowledges, but also to support reflection on how to practice intuition as good care. In order to do so, lessons can be taken from the practice and knowledge of practitioners on the ground. When intuition is acknowledged as a situated practice and CYHC-practitioners are supported in its use through guidelines, chances of detecting child abuse early could increase.

## Supplementary information

**Additional file 1:.** Overview of codes. Overview of codes. A table showing overarching themes, subthemes and codes used in analysis

**Additional file 2:.** Survey questions. Survey questions. A translation of our survey, originally conducted in Dutch

## Data Availability

Data sharing is not applicable to this article as no datasets were generated or analysed during the current study. The interviews collected are in Dutch, but can be provided upon written request. Additionally, upon written request, the data collected via survey can be made available as an SPSS file.

## Abbreviation

CYHC: Child and Youth Health Care

## Footnotes

1 In the Netherlands, the child and youth healthcare system (*jeugdgezondheidszorg*) encapsulates practices related to social work and child protection services. CYHC-practitioners include teams of specialist physicians (*jeugdarts*), nurses (*verpleegkundigen, jeugdverpleegkundigen*) and (physician)-assistants ((*dokters-)assistenten* and *consultatiebureau*-*assistenten*).

2 Different synonyms for intuition discussed with participants, enabling them to pick the term that covered the subject according to them and that they felt most comfortable with. ‘Intuition’ (intuïtie), ‘gut feeling’ (onderbuikgevoel) and a ‘sense of alarm or reassurance’ (pluis/niet-pluis gevoel) were chosen most often. Other words mentioned included ‘fingerspitzengeful’, ‘sensitivity’, ‘subjective feeling’, ‘compass’, ‘sense of alarm’, ‘feeling that something is not okay or off’ and ‘benchmark’ (ijkpunt). Interviewees were asked about their definitions of the synonyms that they were using, to ensure that there was a common understanding.
