# Supplementary material for: Navigating complexity of child abuse through intuition and evidence-based guidelines: a mix-methods study among child and youth healthcare practitioners
Source: BMC Fam Pract. 2020 Aug 1;21:157. doi: 10.1186/s12875-020-01226-6 (PMC7395977; doi:10.1186/s12875-020-01226-6)
Supplement: Supplementary file 1 — Additional file 1:. Overview of codes. Overview of codes. A table showing overarching themes, subthemes and codes used in analysis [file 12875_2020_1226_MOESM1_ESM.pdf]

**Additional file 1: Overview of codes**

A table showing overarching themes, subthemes and codes used in analysis

Table 2: Overview of codes

| **Themes** | **Subthemes** | **Codes** |
|---|---|---|
| *Context* | CYHC-system | Organisations |
| | | Actors |
| | | Hierarchies |
| | | Interests |
| | | |
| | CYHC-practitioners | Education |
| | | Team |
| | | Daily work |
| | | Communication |
| | | Types of cases/families |
| | | |
| | Child abuse | Policies |
| | | Tasks |
| | | Imaginaries |
| | | Decision-making/choices |
| | | Examples |
| | | Fears of CYHC-practitioners |
| | | Fears of families |
| | | |
| *Definitions and recognition intuition* | Words used | Synonyms |
| | | Translations |
| | | Examples |
| | | |
| | Definitions in.. | Practice |
| | | Education |
| | | Guidelines |
| | | Discussions |
| | | |
| | Recognition | Frequency |
| | | Types of situations |
| | | Sensations |
| | | |
| *Attitudes towards intuition* | | Opinions respondent |
| | | Opinions colleagues |
| | | Experiences |
| | | Risks |
| | | |
| | Guidelines | Support |

| | | |
|---|---|---|
| | | Usefulness |
| | | Criticism |
| | | |
| | Intuition versus | Evidence-based medicine |
| | | Subjectivity/Objectivity |
| | | Facts |
| | | |
| *Use in practice* | When? | Experience |
| | | Change over time |
| | | Triggers |
| | | Colleagues |
| | | Examples |
| | | |
| | Normal/abnormal | Norms and values |
| | | Averages |
| | | Risks |
| | | Changes over time |
| | | |
| | Responding to intuition | Decision-making |
| | | Consequences |
| | | Advice guidelines |
| | | Checking intuition |
| | | |
| | Communication | Family |
| | | Colleagues |
| | | Narratives |
| | | Networks of family |
| | | Weighing information |
