# Supplementary material for: Navigating complexity of child abuse through intuition and evidence-based guidelines: a mix-methods study among child and youth healthcare practitioners
Source: BMC Fam Pract. 2020 Aug 1;21:157. doi: 10.1186/s12875-020-01226-6 (PMC7395977; doi:10.1186/s12875-020-01226-6)
Supplement: Supplementary file 2 — Additional file 2:. Survey questions. Survey questions. A translation of our survey, originally conducted in Dutch [file 12875_2020_1226_MOESM2_ESM.pdf]

Additional file 2: Survey questions

Note: this survey was conducted in Dutch, of which a translation is presented below.

Introduction

Thank you for taking part in this survey. First of all, we would like to ask about your work experience, followed by some questions about your opinion with regard to gut feeling. Completing this survey will take approximately 5 minutes. This questionnaire is part of a larger study into the decision-making process of child and youth healthcare pracitioners, initiated by VU University Amsterdam. All your personal data will remain anonymous.

Q1 What is your gender?

Female
Male
Other
No answer

Q2 What is your current position within the youth healthcare system? (you can choose multiple answers)

❑ Consultation clinic assistant
❑ Nurse
❑ Physician-assistant
❑ Child and youth healthcare physician
❑ Staff physician
❑ Attention Officer Child Abuse
❑ Policy advisor
❑ Other answer: ____________________

Q3 How many years have you been working in the youth healthcare system? (Please formulate your answer in full years)

Q4 Are you familiar with gut-feeling during your daily work practices within youth healthcare? A definition of gut-feeling is:

Gut-feeling: the intuitive feeling that something is or is not right, without an (immediate) logical reason.

Yes
No
I don't know

Condition: If No is selected. Skip to: To what extend do you agree with the following propositions. (Q15)
Condition: If Yes is selected. Skip to: To what extent/ How often do you experience gut-feeling? (Q5)

Condition: If I don’t know is selected. Skip to: To what extent/ How often do you experience gut-feeling? (Q5)

Q15 To what extent do you agree with the following propositions?

Gut-feeling: the intuitive feeling that something is or is not right, without an (immediate) logical reason.

	Completely disagree (1)	disagree (2)	Neutral (3)	Agree (4)	Completely agree (5)
1. Sometimes, I come across cases where something isn’t right. I distrust the situation of this client/family.					
2. I sometimes come across cases where I’ve an indefinable feeling, because I’m worried about a possible unfavourable outcome.					
3. Colleagues to whom I look up use their gut feeling when making decisions.<br><br>*For example the decision to make a follow-up appointment, to wait and see, or to discuss the case with a colleague.*					
4. I trust my colleagues when they make decisions based on their gut feeling.<br><br>*For example the decision to make a follow-up appointment, to wait and see, or to discuss the case with a colleague.*					

Q18 To what extent do you agree with the following propositions?

Gut feeling: the intuitive feeling that something is or is not right, without an (immediate) logical reason.

'Decisions' refers to the following: The decision to make a follow-up appointment, to wait and see, or to discuss the case with a colleague.

	Completely disagree (1)	disagree (2)	Neutral (3)	Agree (4)	Completely agree (5)
1. I only make decisions based on facts.<br><br>2. I'm expected to use my gut feeling when making decisions.<br><br>3. Gut feeling is too subjective to use when making decisions regarding (suspected cases of) child abuse or neglect.<br><br>*Subjective: Something is judged or seen from a personal perspective. It involves personal preference or taste. When someone answers subjectively, the answer is influenced by his/her own opinions and experiences. Thus the answer is formed by experiences the person him/herself has been through (Based on the definition by Merriam-Webster dictionary).*					

Condition: Gut-feeling is too sub… Is Selected. Skip To: End of Survey. Condition: Gut-feeling is too sub… Is Selected. Skip To: End of Survey. Condition: Gut-feeling is too sub… Is Selected. Skip To: End of Survey. Condition: Gut-feeling is too sub… Is Selected. Skip To: End of Survey. Condition: Gut-feeling is too sub… Is Selected. Skip To: End of Survey.

Q5 How often do you experience gut-feeling in cases in which you suspect child abuse or -neglect?

Very often
Often
Sometimes
Seldom
Very seldom
Never

Q6 I find the use of gut feeling when making decisions concerning (suspected cases of) child abuse….

For example the decision to make a follow-up appointment, to wait and see, or to discuss the case with a colleague.

	Pleasant (1)	Fairly pleasant (2)	Neutral(3)	Fairly unpleasant (4)	Unpleasant (5)
Pleasant or Unpleasant?					

Q9 I find the use of gut feeling when making decisions concerning (the suspicions of) child abuse….

For example the decision to make a follow-up appointment, to wait and see, or to discuss the case with a colleague.

	Easy (1)	Fairly easy (2)	Neutral (3)	Fairly difficult (4)	Difficult (5)
Easy or Difficult?					

Q11 I find the use of gut feeling when making decisions concerning (the suspicions of) child abuse….

For example the decision to make a follow-up appointment, to wait and see, or to discuss the case with a colleague.

	Good (1)	Fairly good (2)	Neutral (3)	Fairly Bad (4)	Bad (5)
Good or Bad?					

Q12 I find the use of gut feeling when making decisions concerning (the suspicions of) child abuse….

For example the decision to make a follow-up appointment, to wait and see, or to discuss the case with a colleague.

	Useful (1)	Fairly useful (2)	Neutral (3)	Fairly useless (4)	Useless (5)
Useful of Useless?					

Q13 To what extent do you agree with the following propositions?

The word 'decisions' refers to the following: The decision to make a follow-up appointment, to wait and see, or to discuss the case with a colleague.

	Completely disagree (1)	disagree (2)	Neutral (3)	Agree (4)	Completely agree (5)
1. Colleagues to whom I look up use their gut feeling when making decisions.					
2. I'm expected to use my gut feeling when making decisions.					
3. I can trust my gut feeling when making decisions.					
4. I am allowed to use my gut feeling when making decisions.					
5. I decide for myself if I use my gut feeling when making decisions.					

Q20 Thank you for completing the survey! Your input is highly appreciated. All your personal data will remain anonymous.
